# Supplementary material for: Impact of COVID-19 on Urology Practice: A Global Perspective and Snapshot Analysis
Source: J Clin Med. 2020 Jun 3;9(6):1730. doi: 10.3390/jcm9061730 (PMC7356721; doi:10.3390/jcm9061730)
Supplement: Supplementary file 1 [file jcm-09-01730-s001.zip › Suppl1- Appendix A - SIU survey.docx]

**Appendix 1: Complete Survey**

**SIU Survey**

**Urology in the Time of COVID-19**

The Société Internationale d’Urologie (SIU) invites you to complete the following survey on the management of urological conditions in the era of COVID-19. We plan to share the data with our colleagues all over the world to inform them about the current attitudes and practice patterns. This questionnaire will be very helpful.

On behalf of SIU, we sincerely thank you for your collaboration.

# A. Demographic Questions

1. Age:
   - <40 years
   - 40-55 years
   - >55 years
2. In which country are you practicing?

_________________________________

1. Which **one** of the following practice settings best describes your practice?
   - Academic/University Hospital
   - Public non-Academic Hospital
   - Military or Veterans Hospital
   - Private Practice (Office and/or Hospital)

# B. Practical Changes

1. At present, the consultations at the outpatient clinic/office are:
   - Completely locked down
   - Replaced by phone/tele-consultation
   - Restricted only to follow-ups
   - A combination of restricted follow-ups plus phone/tele-consultation
   - Fully operational (no change)
2. In the case of an emergency that requires immediate surgery (e.g., renal injury, trauma), how do you treat?
   - As in the past, without special precautions
   - Assume COVID-19 positive
   - You send a test and proceed without special precautions
   - Increased attention but no specific measures
   - I do not know/not applicable
3. How do you sterilize your surgical equipment?
   - As in the past, without special precautions
   - According to the COVID-19 protocol of our hospital
   - We use single use instruments (ureteroscopes, cystoscopes, laparoscopic equipment) whenever possible
   - I do not know/not applicable
4. How much has the COVID-19 crisis decreased your access to the operating room for elective surgery?
   - No changes
   - Elective surgery reduced by <25%
   - Elective surgery reduced by 25-50%
   - Elective surgery reduced by 50-75%
   - Elective surgery reduced by >75%
   - There is currently no elective surgery
5. Who decides which patients should be operated on in the COVID-19 era at your hospital?
   - The responsible Urologist
   - The Chairman of the Department of Urology
   - A committee of the Surgical Division
   - The Chairman of the Board of the Hospital
   - I do not know/not applicable
6. Elective operations that may require transfusion are:
   - Postponed
   - Performed if patients are at high risk of disease progression
   - Replaced by minimally invasive therapies if possible
   - Performed as in the past (no change)
7. Elective operations that may require admission to the Intensive Care Unit are
   - Postponed
   - Performed if patients are at high risk of disease progression
   - Replaced by minimally invasive therapies if possible
   - Performed as in the past (no change)
8. How do you manage the following?

|  | **Postpone** | **Performed as in the past** | **Outpatient/One-day clinic (under local anesthesia/single use instruments/alternative MITs)** | **Include in the surgical priority list** | **Refer to another center** |
| --- | --- | --- | --- | --- | --- |
| Prostatic biopsy |  |  |  |  |  |
| Cystoscopy |  |  |  |  |  |
| Removal of a double-J stent |  |  |  |  |  |
| Urodynamics |  |  |  |  |  |
| BPH surgery |  |  |  |  |  |
| TUR-bladder tumor |  |  |  |  |  |
| Stone management (without infection) |  |  |  |  |  |
| Radical prostatectomy |  |  |  |  |  |
| Radical/partial nephrectomy |  |  |  |  |  |
| Nephro-ureterectomy |  |  |  |  |  |
| Radical cystectomy |  |  |  |  |  |
| Benign conditions (varicocele, hydrocele, circumcision, TVTs, etc) |  |  |  |  |  |
| Radical orchidectomy/penectomy |  |  |  |  |  |
| RPLND |  |  |  |  |  |

BPH: Benign Prostatic Hyperplasia

TUR: Transurethral Resection

RPLND: Retroperitoneal Lymph Node Dissection

TVT: Tension-free Vaginal Tape

1. Please rate the **priority** of the following types of elective surgery performed at your hospital (1 = lowest priority; 10 = highest priority)

| Surgery | Lowest priority --------------------------------------------- Highest priority | | | | | | | | | | Do not know  Not applicable |
| --- | --- | --- | --- | --- | --- | --- | --- | --- | --- | --- | --- |
|  | 1 | 2 | 3 | 4 | 5 | 6 | 7 | 8 | 9 | 10 |  |
| BPH surgery |  |  |  |  |  |  |  |  |  |  |  |
| TUR-bladder tumor |  |  |  |  |  |  |  |  |  |  |  |
| Stone management (without infection) |  |  |  |  |  |  |  |  |  |  |  |
| Radical prostatectomy |  |  |  |  |  |  |  |  |  |  |  |
| Radical/partial nephrectomy |  |  |  |  |  |  |  |  |  |  |  |
| Nephroureterectomy |  |  |  |  |  |  |  |  |  |  |  |
| Radical cystectomy |  |  |  |  |  |  |  |  |  |  |  |
| Surgery for benign conditions (varicocele, hydrocele, circumcision, TVTs, etc) |  |  |  |  |  |  |  |  |  |  |  |
| Radical orchidectomy/penectomy |  |  |  |  |  |  |  |  |  |  |  |
| RPLND |  |  |  |  |  |  |  |  |  |  |  |

BPH: Benign Prostatic Hyperplasia

TUR: Transurethral Resection

RPLND: Retroperitoneal Lymph Node Dissection

TVT: Tension-free Vaginal Tape

1. Given the current situation, which of the following applies?
   - I am personally not concerned about being infected by COVID-19
   - I am personally concerned about being infected by COVID-19 at work in the hospital
   - I am personally concerned about being infected by COVID-19 from the community
   - I have been infected by COVID-19
2. How do you continue your medical education in the time of COVID-19? (select all that apply)
   - Internet searching
   - Journals and textbooks (including online versions)
   - Podcast/video or audiotapes
   - Online CME courses/webinars
   - No time/interest for this now
3. Please submit up to three (3) COVID-19 related questions or concerns you would like the SIU Office of Education to answer.

***Thank you for completing the survey.***
