# Supplementary material for: Impact of COVID-19 on Urology Practice: A Global Perspective and Snapshot Analysis
Source: J Clin Med. 2020 Jun 3;9(6):1730. doi: 10.3390/jcm9061730 (PMC7356721; doi:10.3390/jcm9061730)
Supplement: Supplementary file 1 [file jcm-09-01730-s001.zip › Suppl2 - Appendix B -Countries Included.docx]

**APPENDIX 2: Countries Included in Each Region**

- **East/Southeast Asia and nearby regions**: Australia, China, Indonesia, Japan, Malaysia, Myanmar, New Zealand, Philippines, Singapore, South Korea, Taiwan, Thailand, Vietnam
- **West/Southwest Asia and nearby regions**: Armenia, Azerbaijan, Bahrain, Bangladesh, Cyprus, Georgia, India, Iran, Israel, Jordan, Kuwait, Kyrgyzstan, Lebanon, Maldives, Nepal, Oman, Pakistan, Qatar, Saudi Arabia, Sir Lanka, Syria, Turkey, United Arab Emirates, Uzbekistan, Yemen
- **Europe:** Albania, Austria, Belarus, Belgium, Bulgaria, Czech Republic, Denmark, Finland, France, Germany, Greece, Hungary, Ireland, Italy, Kosovo, Latvia, Lithuania, Luxembourg, Moldova, Netherlands, Norway, Poland, Portugal, Romania, Russia, Serbia, Slovakia, Spain, Sweden, Switzerland, Ukraine, United Kingdom
- **Africa:** Algeria, Benin, Burkina Faso, Cameroon, Democratic Republic of Congo, Côte D’Ivoire, Egypt, Ghana, Kenya, Mali, Mauritius, Morocco, Nigeria, Senegal, Somalia, South Africa, Sudan, Tanzania, Togo, Tunisia, Uganda, Zimbabwe
- **North America:** Canada, Costa Rica, Cuba, Dominican Republic, El Salvador, Guatemala, Mexico, Nicaragua, Panama, United States
- **South America:** Argentina, Brazil, Chile, Columbia, Ecuador, Paraguay, Peru, Uruguay, Venezuela
